# Cardiovascular Dysfunction in COVID-19: Association Between Endothelial Cell Injury and Lactate

**DOI:** 10.3389/fimmu.2022.868679

**Published:** 2022-03-23

**Authors:** Kun Yang, Matthew Holt, Min Fan, Victor Lam, Yong Yang, Tuanzhu Ha, David L. Williams, Chuanfu Li, Xiaohui Wang

**Affiliations:** ^1^ Department of Surgery, James H. Quillen College of Medicine, East Tennessee State University, Johnson City, TN, United States; ^2^ Center of Excellence in Inflammation, Infectious Disease and Immunity, James H. Quillen College of Medicine, East Tennessee State University, Johnson City, TN, United States; ^3^ James H. Quillen College of Medicine, East Tennessee State University, Johnson City, TN, United States; ^4^ College of Arts and Science, New York University, New York City, NY, United States

**Keywords:** COVID-19, aerobic glycolytic metabolism, lactate, endothelial cell, cardiovascular dysfunction, HMGB1 (High mobility group box 1), thrombosis, vascular permeability

## Abstract

Coronavirus disease 2019 (COVID-19), an infectious respiratory disease propagated by a new virus known as Severe Acute Respiratory Syndrome Coronavirus-2 (SARS-CoV-2), has resulted in global healthcare crises. Emerging evidence from patients with COVID-19 suggests that endothelial cell damage plays a central role in COVID-19 pathogenesis and could be a major contributor to the severity and mortality of COVID-19. Like other infectious diseases, the pathogenesis of COVID-19 is closely associated with metabolic processes. Lactate, a potential biomarker in COVID-19, has recently been shown to mediate endothelial barrier dysfunction. In this review, we provide an overview of cardiovascular injuries and metabolic alterations caused by SARS-CoV-2 infection. We also propose that lactate plays a potential role in COVID-19-driven endothelial cell injury.

## Introduction

Coronavirus disease 2019 (COVID-19) is defined as an infectious respiratory disease propagated by a new virus labeled the Severe Acute Respiratory Syndrome Coronavirus-2 (SARS-CoV-2). COVID-19 was first identified in Wuhan, China in November of 2019 and has become a global health threat affecting more than 200 million people with a mortality rate of 2.3% due to its high contagiousness and lack of specific antiviral treatments (1). There are excellent reviews and articles on the clinical manifestation, hematology laboratory, and management of COVID-19 patients (1–3). By November 2021, over twenty COVID-19 vaccines have been approved in different parts of the world (4). Despite the COVID-19 vaccine, which has been quickly and successfully developed and employed to fight against the COVID-19 infection, the exact mechanisms by which the SARS-CoV-2 significantly causes dysfunction of several systems, including respiratory system, nerve system, and cardiovascular system have not been elucidated entirely.

Emerging clinical data has shown that the COVID-19 patients with cardiovascular diseases (CVDs) have a greater mortality (11%) than in total case mortality (2.3%) (5). On the other hand, COVID-19 infected patients exhibit cardiovascular disorders and heart attack symptoms (6–8). This evidence suggests that SARS-CoV-2 infection could cause cardiovascular dysfunction which is a major factor contributing to the mortality of a substantial proportion of the patients with severe COVID-19 infection. Importantly, the symptoms of severe COVID-19 infected patients resemble the clinical features of endothelial dysfunction (8, 9), indicating that SARS-CoV-2 could cause the endothelium damage. Indeed, electron microscopy analysis of post-mortem tissues showed that SARS-CoV-2 could infect pulmonary endothelial cells and induce endotheliitis in COVID-19 infected patients who are critically ill (9). In addition, SARS-CoV-2 could directly infect endothelial cells *via* angiotensin-converting enzyme 2 (ACE2), subsequently alter the vascular homeostasis, and induce clinical manifestations such as acute respiratory distress syndrome (ARDS) (9–11).

Recent studies highlight the role of metabolisms in the regulation of innate immune and inflammatory responses (12–15). Metabolic reprograming plays a critical role in innate immune and inflammatory responses (16). Severe COVID-19 infected patients usually exhibit the “cytokine storm”, indicating that the metabolisms in these patients have been altered. It is possible that aerobic glycolytic metabolism could be involved in the pathogenesis of the COVID-19 infection that induces severe conditions in those who are infected (17). Importantly, aerobic glycolytic metabolism not only regulates innate immune response (12), but also modulates endothelial cell function (14, 18). Generally, virus infections activate several immune cell types, such as dendritic cells, neutrophils and macrophages to produce pro-inflammatory cytokines that fight against virus invasions and maintain the tissues’ homeostasis (19). This process requires a rapid energy production to provide fuel for immune cell proliferation and inflammation (20). Recent evidence has shown that aerobic glycolytic metabolism and subsequent lactate production can be considered as an integral part of cellular signaling (12–14, 21). Furthermore, more recent findings in immunometabolism show that aerobic glycolysis can be a metabolic choice of immune cells and the function of aerobic glycolytic metabolism is not limited to supporting cell proliferation (22). Thus, it appears that cells can modulate their metabolism to adapt to different energy requirements and signaling events in pathophysiological situations.

Historically, lactate was the end product of aerobic glycolytic metabolism and was considered as a “waste” to be cleared from blood by the liver and kidney (23). Growing evidence suggests that lactate can be used as a sensitive and independent biomarker for critical illnesses, including sepsis (24), cardiovascular dysfunction (25, 26) and various types of cancer (27). Lactate could be a potent signaling molecule in vascular homeostasis, which is supported by a study showing that lactate disrupts vascular barrier function and increases vascular permeability of bone marrow during inflammation (14). Moreover, lactate dehydrogenase (LDH), a key enzyme in aerobic glycolysis, has been associated with worse outcomes in patients with viral infections, including COVID-19 (28–31). In addition, serum lactate levels in severe COVID-19 infected patients are significantly increased (32–35), suggesting increased aerobic glycolytic metabolism in COVID-19 infected patients (17). The important question is whether the COVID-19 infection alters cellular metabolisms that contribute to cardiovascular dysfunction. Based on current knowledge that aerobic glycolytic metabolism is involved in metabolic immune function (36) and cardiovascular dysfunction (37), understanding of the potential mechanisms by which SARS-CoV-2 causes endothelial cell barrier dysfunction could provide preventative and therapeutic solutions for severe COVID-19 patients. In this review, we summarize the association between the COVID-19 infection and cardiovascular dysfunction and discuss the potential role of aerobic glycolytic metabolism and SARS-CoV-2 induced endothelial cell barrier dysfunction, leading to multiple organ damage.

## Cardiovascular Disorder in COVID-19 Patients

### Cardiovascular Disease Is a High Risk for COVID-19 Infection

Cardiovascular disease (CVD) is a common comorbidity in the patients with Severe Acute Respiratory Syndrome (SARS) and was just as prevalent in patients who experienced Middle East Respiratory Syndrome (MERS) during the previous global pandemic (38). Numerous studies have shown that there are similar genetic identity (79.6%) and biological features shared between SARS-CoV-2 (for COVID-19) and SARS-CoV (for SARS) (39–41). One study that was conducted held a 12-year follow-up that consisted of 25 patients who had recovered from the SARS-CoV infection; among these patients, 44% of them exhibited cardiovascular system abnormalities (42). Once again, due to the similarity in structure between SARS-CoV and SARS-CoV-2, it is highly possible that Covid-19 may also cause similar future troubles for the myocardium. Therefore, it is no surprise that CVD is present in the patients with COVID-19 (with a prevalence of ~17%) (6, 43, 44). A study, including 416 hospitalized COVID-19 infected patients in Wuhan (China), by Shi et al. showed that patients with a history of CVDs had higher risk of in-hospital death (45). Similarly, reports involving 1,591 patients (with a mortality rate of 26%) with COVID-19 in the Lombardy (Italy) (46) and 393 patients with COVID-19 in New York City (USA) (47) showed pre-existing CVD rates of 21% and 14%, respectively. In addition, a meta-analysis of fifty-six studies including 159,698 COVID-19 patients revealed that 25% of ICU-admitted patients had CVD, and the pooled prevalence of acute cardiac injury by 33.6%, arrhythmia by 33.0%, heart failure by 20.4%, coronal artery disease 20.6% and hypertension by 43.6%, respectively (48). Another meta-analysis including both ICU and non-ICU COVID-19 infected patients in China (1,527 cases in total) showed that the proportions of hypotension, cardio-cerebrovascular disease and diabetes in COVID-19 patients were 17.1%, 16.4% and 9.7%, respectively (49). Among these patients, the incidences of hypertension, cardio-cerebrovascular disease and diabetes were at least twofold higher in ICU cases than in non-ICU counterparts (49), indicating that the patients with CVD are more susceptible to suffer severe condition and are at a higher risk of death. In a study from the National Health Commission of China (NHC), mortality data for Covid-19 was released, and it determined that 17% of the patients exhibited a history of coronary heart disease while 35% had a history of hypertension (50). These data led to the conclusion that who had any sort of underlying CVDs and were simultaneously infected with SARS-CoV-2 had a higher chance of experiencing more severe symptoms. Therefore, it can be inferred that CVD/CVD-related risk factors strongly affect the prognosis of the COVID-19 patients.

### COVID-19 Infection Induces Cardiovascular Dysfunction

Importantly, COVID-19 infected patients who do not have CVD exhibit cardiovascular dysfunctions, including myocardial injury, cardiac arrhythmia, as well as thrombotic complications; this indicates that COVID-19 itself can induce cardiovascular disorders (6, 51). This mechanism of direct infection occurs when the virus immediately infects cardiomyocytes originating from induced pluripotent stem cells (iPSCs); as a result, SARS-CoV-2 infection in iPSCs induces morphological and cytotoxic effects characterized by detaching from neighboring cells and increased cell death, suggesting SARS-CoV-2 directly causes damages to cardiac tissue (52). This observation may explain myocardial complications in SARS-CoV-2 infection. Furthermore, in another study pertaining to iPSCs, data revealed that after 72 hours of exposure to the SARS-CoV-2 infection, apoptosis as well as cessation of beating will appear (53). To measure the severity of injury done to the myocardium, the use of serum troponin (troponin T or troponin I) level, which is a specific marker for cardiac injury, can be applied (54–56). A multicenter study showed that 278 (45.3%) of 614 COVID-19 infected patients had elevated serum levels of troponin (troponin T or troponin I) (56). Importantly, increased troponin levels, independent from concomitant cardiac disease, were associated with increased in-hospital mortality (56). A report from the National Health Commission of China showed that serum troponin I levels were increased and cardiac arrest occurred in 12% of COVID-19 infected patients who did not have CVDs previously during hospitalization (2, 44, 50). Data from autopsy analysis shows that SARS-CoV-2 virus was identified in 24 (61.5%) cardiac tissues of 39 patients with COVID-19 infection (57). A report from a single-center shows that cardiac arrhythmia was also a prevalent manifestation in 138 patients with COVID-19 infection (58). This study documented that arrhythmia occurred in 17% of hospitalized patients and 44% of ICU-admitted COVID-19 infected patients (58). With a broad range of laboratory coagulation parameter alterations including D-dimer, prothrombin time and fibrinogen in COVID-19 infected patients, coagulation dysfunction has been considered as a hallmark of SARS-CoV-2 infection. Tang et al. observed that, in 183 consecutive COVID-19 infected patients, non-survivors had higher D-dimer levels, fibrinogen degradation products and longer prothrombin time, when compared with survivors (59). A study by Klock et al. shows that the incidence of thrombotic complications was 31% in 184 ICU-admitted patients with COVID-19 infection in Dutch (60). This study also shows that venous thromboembolism, confirmed by CT pulmonary angiogram (CTPA) and ultrasonography, accounted for 87% of all thrombotic events (60). The thromboembolism in COVID-19 infected patients may result from excessive inflammation, hypoxia and diffuse intravascular coagulation (61, 62). However, anticoagulant therapy using prophylactic heparin in COVID-19 patients who developed sepsis-induced coagulopathy markedly reduced 28-day mortality from 64% to 40% (63). In addition, uncontrolled blood pressure is a risk factor for COVID-19 patients by causing acute kidney injury and chronic obstructive pulmonary disease (COPD) (64).

## Endothelial Cell Dysfunction in COVID-19 Infected Patients

The endothelium is a layer of endothelial cells (ECs) that line the interior surface of blood vessels and plays a critical role in mediating vasomotor tone, maintaining blood fluidity, and balancing local inflammatory mediators (65, 66). The maladaptive response of ECs to acute inflammation contributes to the pathogenesis of various infectious diseases and multiple organ dysfunction syndrome (MODS) (67). Several recent studies investigated mechanisms by which SARS-Cov-2 induces EC dysfunctions, including inflammation, vasoconstriction, permeability, and coagulation (68–70). A study by Ackermann et al. shows that COVID-19 infection not only causes acute respiratory distress syndrome (ARDS), but also harms the vasculature (71). This pathologic study included seven lung tissues from COVID-19 infected patients, seven lungs from Influenza (H1N1) patients with ARDS, and 10 from age-matched uninfected controls. These lung tissues were examined with seven-color immunohistochemical analysis, micro-computed tomographic imaging, scanning electron microscopy, corrosion casting, and direct multiplexed measurement of gene expression. The authors compared the results between the groups focusing on the three distinct angiocentric features including: 1) severe endothelial injury associated with intracellular SARS-CoV-2 virus and disrupted endothelial cell membranes, 2) widespread vascular thrombosis with microangiopathy and occlusion of alveolar capillaries, and 3) significant new vessel growth through a mechanism of intussusceptive angiogenesis. The COVID-19 infected patients exhibited 9 times more alveolar capillary microthrombi (P<0.001) compared with H1N1 influenza patients. COVID-19 infected patients also presented with 2.7 greater of the amount of new vessel growth through intussusceptive angiogenesis (P<0.001) than in H1N1 influenza. In addition, endothelial cells from the COVID-19 patients exhibited cellular swelling, disrupted intracellular junctions, and a loss of contact with the basement membrane. An *in vitro* study by Robles and colleagues showed that the spike protein of SARS-CoV-2 promotes the expression of leukocyte adhesion molecules VCAM1 and ICAM1 upon binding to integrin α5β1 on ECs, resulting in increased leukocyte adhesion to ECs (72). A previous study shows that integrin α5β1 activates NF-κB in ECs to elicit inflammation (73). Consistently, SARS-CoV-2 protein treatment enhanced p65 nuclear accumulation and IL-6 expression in ECs (72, 74). To explore the mechanism of myocardial injury in COVID-19 infected patients, Feng et al. utilized a rhesus macaque model of SARS-CoV-2 respiration tract infection (75). They observed that increased infiltration of inflammatory cells in left ventricle tissues and elevated levels of inflammatory cytokines in infected macaques, suggesting the occurrence of viral myocarditis following SARS-CoV-2 infection (75). Notably, the expression of ICAM1 and VCAM1 in the left ventricle tissues was also upregulated in infected macaques as compared to healthy controls (75). These findings provided evidence showing that the endothelial cell damage may be attributed to direct SARS-CoV-2 infection and perivascular inflammation.

## ACE-2 and Endothelial Cell Dysfunction in COVID-19 Infection

Angiotensin-Converting Enzyme 2 (ACE2) is a type-I transmembrane glycoprotein that negatively regulates the renin-angiotensin system (RAS) by degrading Ang II to the heptapeptide Ang 1-7 (76, 77). The protein was initially identified as a homolog to ACE in 2000 by Tipnis et al. (78). Besides its peptidase-dependent actions in regulating RAS, ACE2 was then identified as an essential receptor for SARS coronavirus in 2003 (79). Although ACE2 is shown as a protective molecule against lethal lung injury in SARS, the expression of ACE2 is not limited to respiratory system (80). Instead, a recent immunohistochemical analysis showed that ACE2 has limited expression in respiratory tracts compared to other tissues/cells, including enterocytes, renal tubules, gallbladder, cardiomyocytes, male reproductive cells, placental trophoblasts, ductal cells, eye, and vasculature (76). Intriguingly, ACE2 is expressed in arterial and venous endothelial cells and arterial smooth muscle cells in various human organs (81). It is suggested that ACE2 is required to maintain the endothelial integrity inside the vessels (11). Indeed, existing data for SARS-CoV-1 in 2002 SARS pandemic indicate that virus binding can reduce ACE2 levels, which may lead to endothelial dysfunction (80).

Recent evidence suggests that ACE2 is a functional receptor for SARS-CoV-2 to enter host target cells (82, 83). The infection of SARS-CoV-2 begins with SARS-CoV-2 cleaving its S protein through transmembrane protease serine 2 (TMPRSS-2) and attaching to the ACE-2 receptor (84–86). This ongoing infection produces significant endotheliitis, a robust immune response and a subsequent increase in pro-inflammatory cytokines, vasoactive molecules, and immune cells like neutrophils, macrophages, monocytes, and lymphocytes which all play a role in propagating a response known as the cytokine storm (71, 84, 87, 88). *In vitro* study using engineered human blood vessel organoids showed that SARS-CoV-2 can directly infect endothelial cells *via* ACE2 (89). Varga et al. reported that the presence of viral inclusion structures was detected in endothelial cells in COVID-19 patients (9). Intriguingly, neutralization of ACE2 using soluble human ACE2 decreased virus-infected endothelial cells *in vivo* (89). These pieces of evidence highlight the role of ACE2 in mediating of SARS-CoV-2 induced endothelial cell injury. Indeed, the endothelium is a vulnerable target by SARS-CoV-2 infection, and these infected endothelial cells exhibit dramatic changes in morphology and function (71). Therefore, endothelial cell dysfunction could be an important and potential pathogenesis of COVID-19 infection induced multiple organ dysfunction. In the following sections, we step beyond our focus on the virus and discuss the role of aerobic glycolytic metabolism in COVID-19-driven endothelial cell injury in order to better understand the potential mechanisms that cause the endothelial cell damage in COVID-19 infected patients.

## Switching Metabolism in COVID-19 Infection

Previous studies have shown that virus infection dramatically modified the cellular metabolism of host cells (90). It is hypothesized that the virus-driven metabolic process in a host cell is to provide macromolecules needed for virion replication and assembly (20, 90). Thomas et al. observed that the levels of glucose and free fatty acid in the serum of COVID-19 infected patients were significantly increased providing fuels for viral proliferation (91). Similarly, Shen et al. reported that the serum glucose levels were elevated in the severe patients infected with COVID-19, when compared with control groups (92). In addition, patients with pre-existing metabolic diseases, including diabetes, have greater risk of developing severe conditions (93). A study including 174 COVID-19 infected patients implies that diabetic patients without other comorbidities were at high risk of severe pneumonia, excessive inflammation responses and hypercoagulable state (94). To understand the potential mechanism by which uncontrolled diabetes is a risk factor for severe COVID-19, Codo et al. investigated the correlation between glycolysis and SARS-CoV-2 replication and found that glucose enhanced SARS-CoV-2 load in monocytes in a dose dependent manner (95). In agreement, a retrospective observational study, including 2433 COVID-19 patients admitted to the Houshen Shan hospital in Wuhan between February and April in 2020, indicates that elevated glucose level could be a predictive maker for the disease progression and the fatality of COVID-19 patients (96). Moreover, He et al. reported that COVID-19 infected patients without pre-existing diabetes also presented high blood glucose levels (97), indicating that SARS-CoV-2 infection may change metabolic profiles in these patients. Indeed, most viruses tested to date can induce aerobic glycolytic metabolism to favor their replication (20), which seems to be the same case for SARS-CoV-2 infection. Thus, when the virus enters a diabetic patient, especially a Type II diabetic patient, the high glucose levels within the host results in a disrupted glucose metabolism. This disruption favors SARS-CoV-2 replication and cytokine production while simultaneously dampening the proper effects of the immune system (T-cell response/function is worsened), prompting a more severe inflammatory response (cytokine storm) within this demographic (95).

Mitochondria are essential cellular organelles in regulating cellular energy, metabolism, and host innate immunity (98–100). Transcriptomic study by Mooamalla et al. shows that SARS-CoV-2 infection downregulated tricarboxylic acid cycle (TCA) and oxidative phosphorylation in several human respiratory cell lines, indicating mitochondrial dysfunction (101). Indeed, emerging evidence shows that SARS-CoV-2 highjacks mitochondria and replicates in mitochondria, leading to impaired mitochondrial dynamics and cell death (102). It is proposed that aerobic glycolytic metabolism is enhanced when mitochondrial defect occurs (103, 104). Mooamalla and colleagues also found that the expression of lactate dehydrogenase (LDHA), which is a dispensable enzyme for aerobic glycolysis, was increased and lactate production was elevated in SARS-CoV-2-infected human respiratory cell lines (101). Notably, similar observation is made in peripheral blood mononuclear cells (PBMCs) isolated from COVID-19 patients, in which the rate of glycolysis was increased, and the mitochondrial respiration was impaired (105).

It has been reported that SARS-CoV-2 affects both the upper and lower respiratory tract, which, in many cases, results in hypoxemia (106, 107). In addition to virus-driven metabolic changes, lack of oxygen may also be a determinant in regulating metabolism in patients with COVID-19 infection. Inadequate oxygen supply shifts oxidative phosphorylation to aerobic glycolysis, leading to increased production of lactate and extracellular acidification. It is demonstrated that lactate is a natural suppressor for antiviral signaling though inhibiting retinoic acid-inducible gene (RIG) (21). Collectively, COVID-19 infection could induce a metabolic switch from oxidative phosphorylation to aerobic glycolysis which does not only benefit to virus replication, but also priming innate immunity mediated pro-inflammatory cytokine production (95, 108). In addition, the intermediates of aerobic glycolytic metabolism could play an important role in the regulation of pro-inflammatory response and endothelial cell dysfunction (12, 14).

## Aerobic Glycolytic Metabolism and Endothelial Cell Injury in COVID-19 Infection

As mentioned above, growing evidence shows that COVID-19 infection switches metabolisms from oxidative phosphorylation to aerobic glycolytic metabolism (17, 105), which allows the rapid production of energy and other substrates for viral replication (20). Lactate is the end product of aerobic glycolysis and serves as an important diagnostic biomarker for critical illnesses, such as sepsis/septic shock (24). It has been shown that severe COVID-19 patients developed typical symptoms that are similar to septic shock, such as vascular microthrombosis, multi-organ dysfunction syndrome (MODS), coagulopathy, high cytokine production (109). Considering the parallels in the pathophysiology of sepsis and COVID-19, it is proposed that viral sepsis is crucial to the pathogenetic mechanisms of COVID-19 (110). In this case, lactate generated from aerobic glycolytic metabolism may be also applied as a biomarker for diagnosis and prognosis of COVID-19 infected patients. Velavan et al. showed that hospitalized patients with moderate to severe COVID-19 (N = 18) had significantly higher blood lactate levels than mild ambulatory COVID-19 patients (N = 16) (33). In addition, a retrospective study including 45 ICU-admitted patients with COVID-19 showed that sequential organ failure assessment (SOFA) score and initial blood lactate levels were significantly higher in non-survivors (N = 11) compared to survivors (N = 34), indicating that blood lactate level mirrors organ dysfunction and is associated with poor clinical outcomes of COVID-19 ICU patients (32). In consistent with this observation, Metkus et al. reported that non-survivors (N = 88) had significantly elevated blood lactate levels than survivors (N = 155) of COVID-19 patients (3.6 mmol/L vs. 2.0 mmol/L, *P* = 0.005) (111). Moreover, blood lactate levels positively and independently correlate with troponin (troponin I or troponin T) levels in COVID-19 patients (N = 243, *P* = 0.007), suggesting that lactate may serve as predictor for myocardia injury in COVID-19 patients (111). These pieces of evidence suggest that elevated lactate levels could correlate with both severity and mortality of COVID-19. Of note, a pooled analysis, including 1,532 COVID-19 patients, reported that increased lactate dehydrogenase (LDH) levels were associated with a 6-fold increase in odds of severe COVID-19 and 16-fold increase in odds of COVID-19 mortality (112). Given that LDH is involved in lactate production, it is advisable that lactate consumption might be also increased.

Although lactate was considered as a waste in the past decades (23), growing evidence has shown that lactate may exert important regulatory roles in various pathophysiological processes, including immunosuppression (12, 113–115), cell signaling (13, 116) and gene transcription (117–119). Recent studies further reveal that lactate can directly induce permeability in inflammatory bone marrow endothelium by downregulation of VE-cadherin expression (14), indicating that lactate could contribute to the pathophysiologic mechanisms of cardiovascular injury in COVID-19 infection. As a result, the implication of serum lactate may be able to present us with an improved method of measuring clinical severity and observe clinical treatment response in the context of COVID-19. In the sections below, we discuss the possible mechanisms of lactate-mediated endothelial injuries in the pathogenesis of COVID-19 infection.

### Lactate and SARS-CoV-2 Infection-Induced Endothelial Cell Injury

The integrity of endothelium is required for maintaining the vascular homeostasis (120). SARS-CoV-2 infects the host cells using the ACEs receptor (85), which is expressed by endothelial cells. Established evidence suggests that SARS-CoV-2 hijacks the endothelial cells and causes significant changes in endothelial cell morphology, *i.e*. disruption of intercellular junctions and cell swelling in COVID-19 infected patients (9, 71). Several lines of *in vitro* and *in vivo* evidence also demonstrate that endothelial cells are highly susceptible to SARS-CoV-2 infection (89, 121–123). It has been reported that SARS-CoV-2 proliferation in endothelial cells directly induces apoptosis in COVID-19 patients (9). In addition, circulating endothelial cells (CECs) have been considered as a marker for damaged endothelium in various vascular diseases (124–126). Importantly, COVID-19 infected patients have higher numbers of CECs than healthy controls, indicating the occurrence of endothelium damages in COVID-19 patients due to direct virus infection (127).

**Figure 1:**
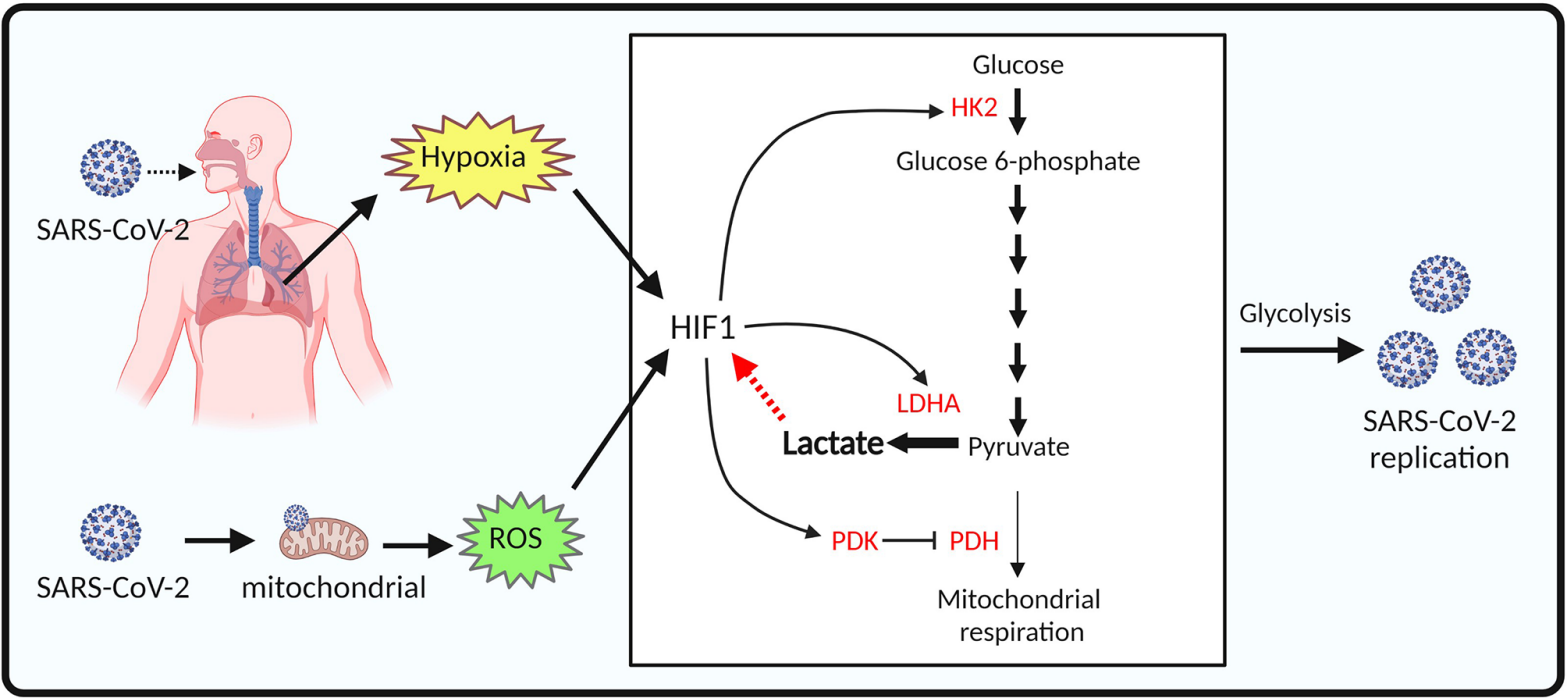
Proposed model of aerobic glycolysis activation in SARS-CoV-2 infected endothelial cells. SARS-CoV-2 infection of pulmonary tissues leads to hypoxia. SARS-CoV-2 infection also causes mitochondrial dysfunction and reactive oxygen species (ROS) production in endothelial cells. Both hypoxia and ROS mediate HIF-1 stabilization. Enzymes involved in glycolysis, including hexokinase (HK), pyruvate kinase 2 (PKM2), lactate dehydrogenase (LDH) are upregulated by HIF-1 signaling, resulting in increased lactate production and SARS-CoV-2 replication in endothelial cells.

Infection of SARS-CoV-2 in the pulmonary tissues impairs gas exchange leading systemic hypoxia and enhanced glycolysis metabolism in endothelial cells and immune cells by stabilizing hypoxia-inducible factor-1 (HIF-1) (128). HIF-1 is a powerful inducer of glycolysis *via* upregulation of enzymes involved in glycolysis, including hexokinase (HK), pyruvate kinase 2 (PKM2), LDHA/LDHB and pyruvate dehydrogenase kinase (PDK) in COVID-19 (129–131) (**Figure 1**). In addition, SARS-CoV-2 infection triggers mitochondrial ROS production, leading HIF1 stabilization and consequently promotes glycolysis (95) (**Figure 1**). Notably, it is proposed that lactate can induce the activation of hypoxia-inducible factor-1 (HIF-1), which further enhances aerobic glycolysis and promotes SARS-CoV-2 infection and replication (95, 132, 133). Indeed, inhibition of lactate production by 2-DG or oxamate suppressed aerobic glycolysis efficiently and reduced viral load in human monocytes (95). Previous studies demonstrate that endothelial cells rely heavily on aerobic glycolysis for ATP production while having little glucose oxidation (134, 135). This may make endothelial cells more susceptible to SARS-CoV-2 infection. Therefore, it is possible that lactate generated from aerobic glycolysis could be beneficial to SARS-CoV-2 proliferation and mediation of endothelial cell injury.

### Lactate and Endothelium Permeability in COVID-19 Infection

Endothelium hyperpermeability contributes to tissue fluid overload (edema) and the persistent hypotension in critically ill patients. Prolonged edema may lead to multiple organ failure and ultimately death (136). Clinical data suggests that COVID-19 infected patients with severe conditions exhibit lower values of serum albumin, indicating the presence of vascular permeability (137). Wu and colleagues provided histological evidence showing that ICU-admitted COVID-19 infected patients who were characterized with hypoalbuminemia had disrupted inter-endothelial junctional complex in the lung tissues (138). It is well known that disarrangement of junctional proteins in the plasma membrane of adjacent endothelial cells increases vascular permeability (139, 140). Vascular endothelial cadherin (VE-cadherin) is one of the determinants of endothelial cell contact integrity (141). A recent study by Flores-Pliego et al. showed that the expression of VE-cadherin, as well as Claudin 5, decreased in the endothelium of decidua and chorionic villi of placentas derived from women with severe COVID-19, when compared to healthy controls (142). Similarly, Feng and colleagues utilized a rhesus macaque model of SARS-CoV-2 respiratory tract infection and observed that SARS-CoV-2 infection significantly reduced VE-cadherin levels in the heart of rhesus macaques when compared to uninfected controls (75). In agreement with these *in vivo* observations, several *in vitro* studies demonstrated that SARS-CoV-2 spike proteins can also disorganize the VE-cadherin complex and decrease VE-cadherin levels in cultured endothelial cells (69, 72, 74). Therefore, SARS-CoV-2 infection can disrupt VE-cadherin largely responsible for the vascular permeability in COVID-19 patients.

**Figure 2:**
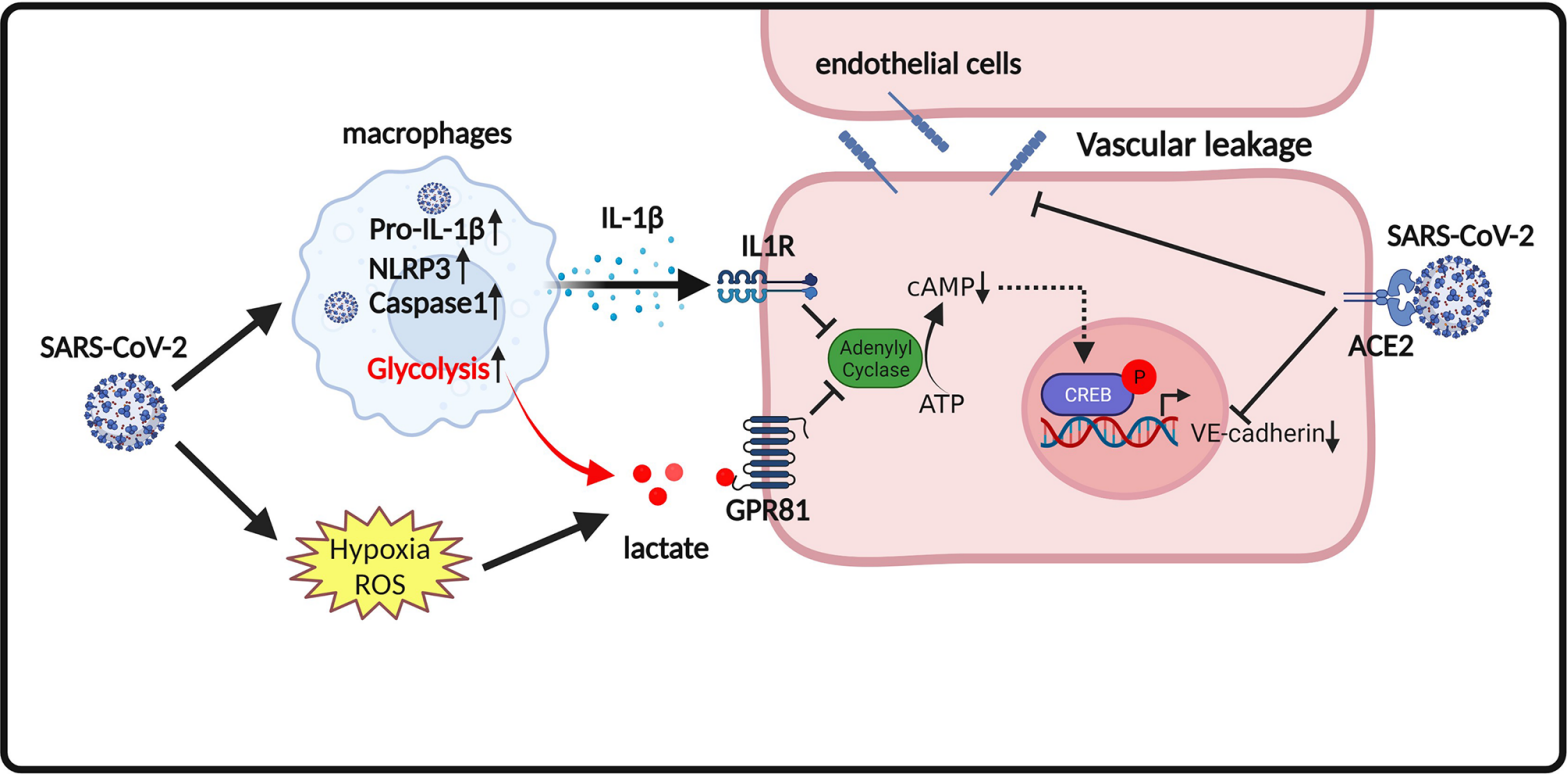
Proposed model of endothelium permeability induced by lactate/GPR81 signaling and SARS-CoV-2 infection. SARS-CoV-2 infection promotes the release of the pro-inflammatory cytokine IL-1β. IL-1β suppresses cAMP formation and CREB-mediated transcription of VE-cadherin in endothelial cells. SARS-CoV-2 infection also increases lactate production. Lactate activates GPR81 and reduces cAMP generation and CREB-mediated transcription of VE-cadherin in endothelial cells. In addition, SARS-CoV-2 spike proteins directly disorganize VE-cadherin complex and suppress VE-cadherin transcription in endothelial cells. Disruption of VE-cadherin complex is responsible for vascular permeability in COVID-19.

Notably, a recent study by Khatib-Massalha et al. showed that lactate directly decreases VE-cadherin expression in endothelial cells, which contributes to the hyperpermeability of bone marrow (BM) endothelium (14). G protein-couple receptor 81 (GPR81) is a lactate receptor (143). Activation of GPR81 by its agonist (3,5-DHBA) has similar effects as lactate on reducing the expression of VE-cadherin in endothelial cells (14). In contrast, knockout of GPR81 attenuated lactate-induced BM vascular permeability, demonstrating that GPR81 is essential for lactate-induced vascular permeability (14). In addition, it is reported that SARS-CoV-2 infection activates pyroptotic signaling in lungs and promotes interleukin-1β (IL-1β) release, which results in downregulation of VE-cadherin on lung endothelial cells (144). IL-1β-induced downregulation of VE-cadherin contributes to lung vascular injury following SARS-CoV-2 infection (144). The underlying mechanism for IL-1β-induced downregulation of VE-cadherin in SARS-CoV-2-infected endothelial cells could be mediated by cAMP response element binding protein (CREB)-mediated suppression of VE-cadherin transcription (145). Therefore, it is conceivable that the action of lactate in promoting vascular permeability is mediated not only by favoring SARS-CoV-2 replication cells, but also by directly disrupting VE-cadherin and suppressing VE-cadherin transcription in endothelial cells upon GPR81 activation (**Figure 2**).

### Lactate and Coagulation in COVID-19 Infection

**Figure 3:**
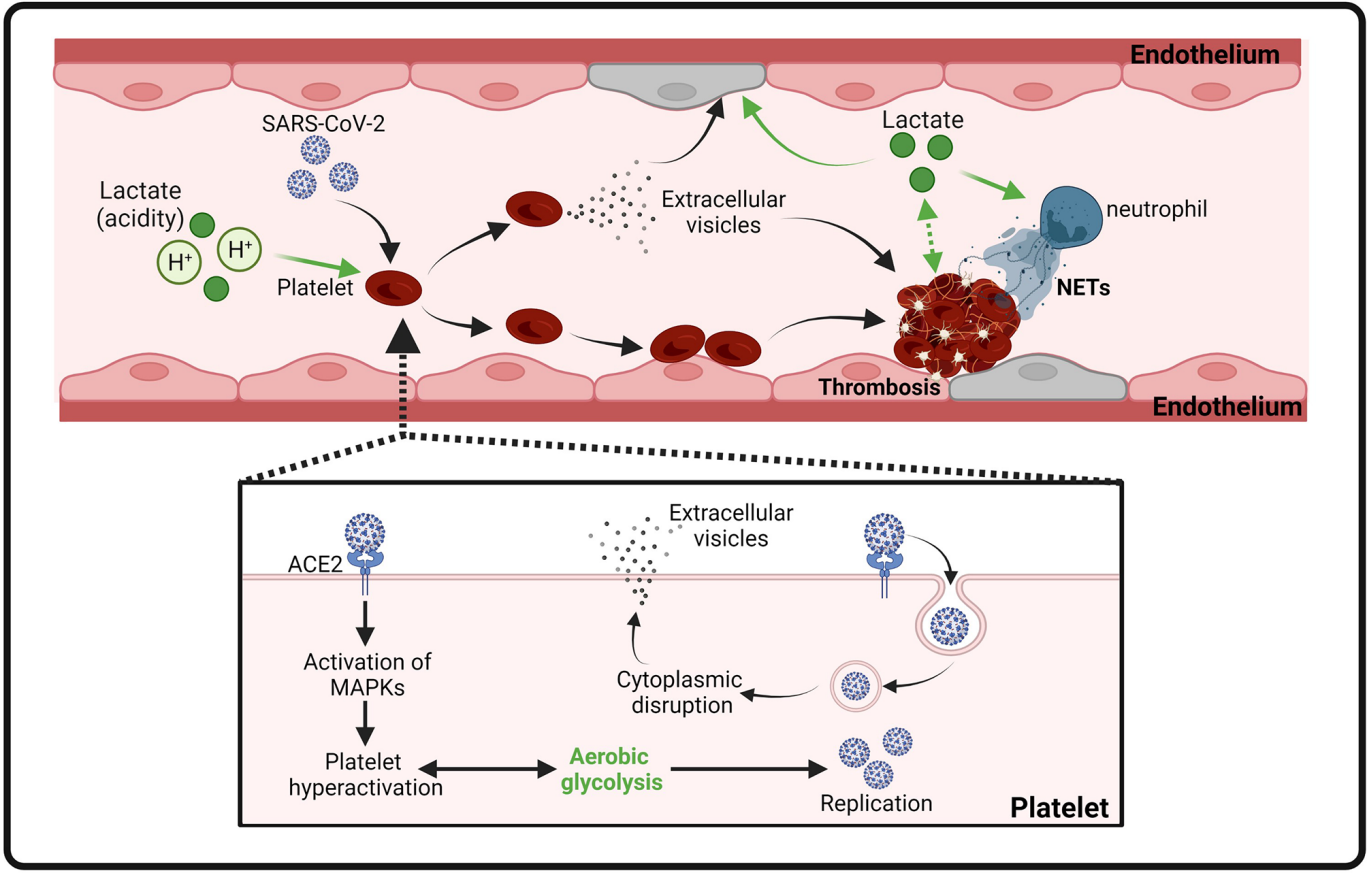
Proposed model of platelet activation, thrombosis and endothelial cell injury induced by SARS-CoV-2 infection and lactate. Binding of SARS-CoV-2 spike protein to ACE2 leads to MAPK signaling activation and subsequent platelet activation. Activated platelets release coagulation factors and cytokines to promote thrombosis. Internalization of SARS-CoV-2 virions induces the release of extracellular vesicles from platelets to facilitate thrombosis. In addition, lactate (acidity) also contributes to thrombosis by promoting activation of platelets, endothelial cells, and NETs.

COVID-19-induced multiple organ damage is associated with an abnormal coagulation (146). COVID-19 patient autopsies have revealed thrombi in the microvasculature (147). All-cause mortality in COVID-19 patients with thrombotic events is significantly higher than those without thrombotic events (148). Several hospital-based studies in Wuhan (China) reveal that some of the COVID-19 patients had elevated serum levels of pro-coagulation factors, including prothrombin (PT) and D-dimer, while the levels of fibrinogen and platelet are normal, representing the risk of thrombosis (59, 149, 150). Mechanistic studies reveal that SARS-Cov-2 spike protein directly binds platelet ACE2 and induces phosphorylation of ERK, p38 and JUK to activate platelets, which promotes thrombosis in COVID-19 (151). In addition, a recent study shows that SARS-Cov-2 virions can be internalized by platelets causing programmed cell death of platelets and extracellular vesicle release from platelets (152) (**Figure 3**). Moreover, inflammation and metabolism changes caused by SARS-Cov-2 infection are also considered as major contributors to coagulopathy in infected patients (153). However, with currently unknown mechanisms, clinical management of thrombosis with standard anti-coagulation dose of heparin failed to show satisfying outcomes (154–156). Two other plausible methods of managing coagulation include RAS inhibitors and statins. It is reported that the implementation of either substance has beneficial effects on COVID-19 clinical symptoms (157–159).

Thachil et al. has recently discussed that hypoxia could be a mechanism of heparin resistance in COVID-19 patients (61). Indeed, oxygen deprivation has long been associated with thrombosis by triggering the pro-coagulation pathway (160). Both hypoxia and infection can result in enhanced aerobic glycolysis and consequent accumulation of lactate. It is noteworthy that metabolome analysis of venous thrombus from rabbits revealed that lactate is one of the most abundant metabolites in the thrombus (161, 162). Activated platelets, together with endothelial cells, are critical mediators of arterial thrombosis (163). Regardless of the nature of their stimulus, activated platelets switch their metabolism to aerobic glycolysis and produces a significant amount of lactic acid (164, 165). Increased extracellular lactate levels and acidity may further induce the continuous activation of Na^+^/H^+^ exchanger (NHE) in platelets and vascular endothelium, leading to the development of thrombosis (166, 167). In addition, elevated lactate levels in pulmonary embolism (PE) patients have been shown to correlate with impaired plasma fibrinolytic capacity and increase thrombin generation and neutrophil extracellular trap (NET) formation (168) (**Figure 3**). Importantly, pharmacological inhibition of aerobic glycolysis, which suppresses lactate production, efficiently reduced thrombosis in mice (165).

### Lactate and HMGB1 in COVID-19 Infected Patients

**Figure 4:**
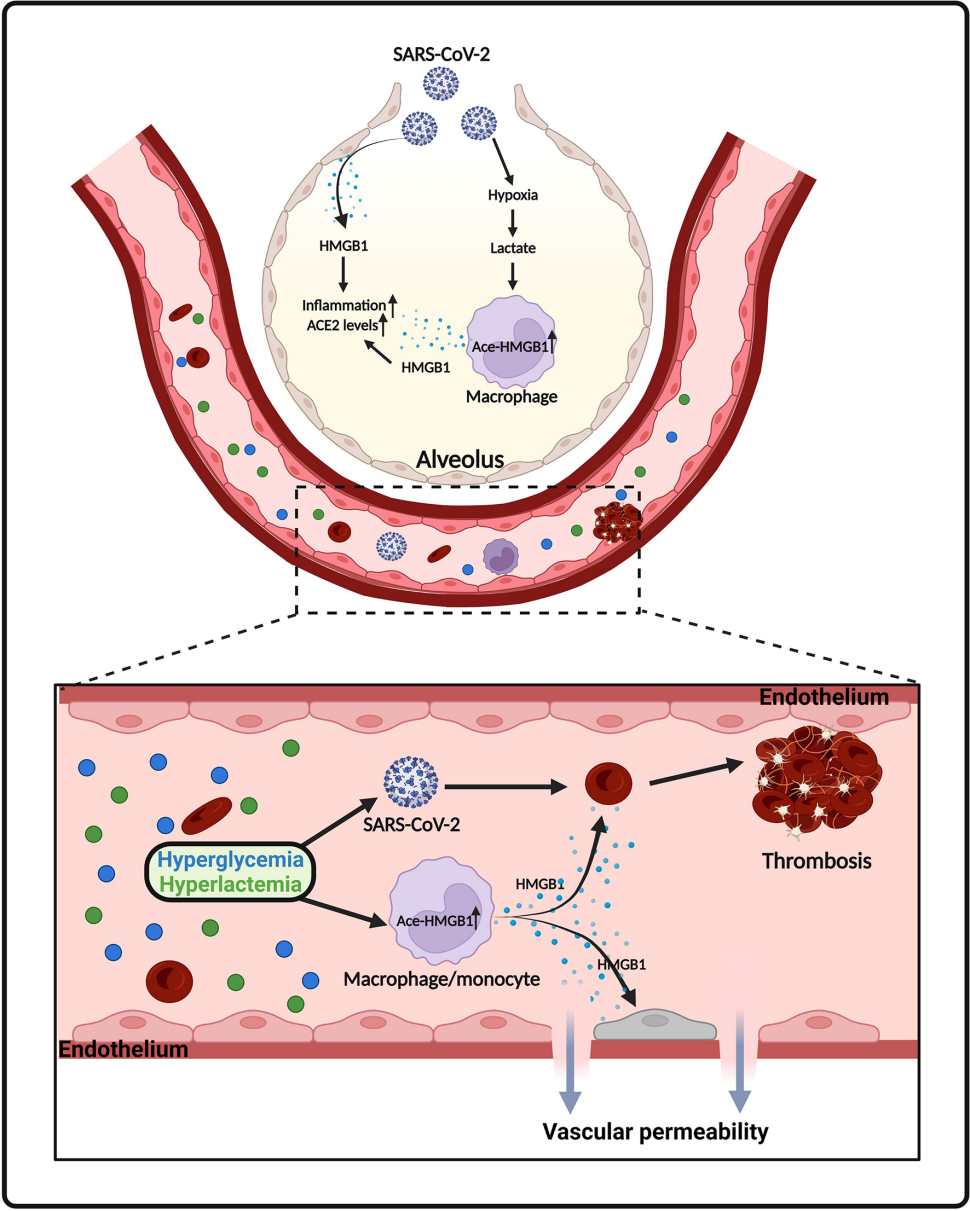
Proposed model of HMGB1 release in SARS-CoV-2 infection. SARS-CoV-2 infection causes death of epithelial cells and release of HMGB1. Lactate, derived from aerobic glycolysis, also promotes HMGB1 acetylation and release from macrophages/monocytes. Elevated levels of HMGB1 further promotes inflammatory responses, ACE2 expression, endothelium permeability and thrombosis in COVID-19.

High mobility group box 1 (HMGB1) is a chromatin-linked small protein that has nuclear, cytosolic and extracellular functions in various pathophysiological processes (169–174). Accumulating evidence shows that serum HMGB1 level is a potential biomarker for COVID-19 infected patients (175). A retrospective study, including 121 COVID-19 patients, shows that circulating HMGB1 and S100A8/A9 levels were significantly elevated in ICU-admitted COVID-19 patients (N = 40) compared to non-ICU COVID-19 patients (N = 81) (176). A similar observation was made by Chen et al. showing that severe COVID-19 patients (N = 11) had significantly higher levels of HMGB1 than non-severe COVID-19 patients (N = 29) (175). Gowda et al. reported that overexpression of SARS-Cov-2 spike protein in respiratory epithelial cells increased HMGB1 levels (52). In addition, SARS-Cov-2 spike protein caused cell death of epithelial cells, which may be responsible for subsequent release of HMGB1 (52) (**Figure 4**).

We and others have shown that adaption to aerobic glycolysis in immune cells promotes the acetylation of HMGB1, leading to its extracellular release during infection (13, 177). HMGB1 acetylation is a concisely regulated process that involves various signaling pathways. Lu et al. shows that activation of JAK/STAT1 signaling is sufficient for LPS-induced HMGB1 hyperacetylation and cytoplasmic accumulation in macrophages (178). In addition, HMGB1 acetylation and release can be regulated by poly(ADP-ribose) polymerase-1 (PARP-1) in activated immune cells (179, 180). Moreover, previous studies indicate that HMGB1 acetylation is part of a general acetylation wave controlled by histone lysine acetylases and deacetylases (181–183). Interestingly, lactate is a potential inhibitor of histone lysine deacetylases (117). Indeed, our recent study demonstrated that lactate significantly increased nuclear translocation of histone lysine acetylases CBP and p300, while suppressed the expression of histone lysine deacetylase SIRT1 in macrophages (13). This regulatory role of lactate tilts the balance of acetylation/deacetylation of HMGB1 towards acetylation (13). Acetylated HMGB1 mainly localized in cytoplasm and subsequently released into the extracellular environment. In an *in vitro* endothelium barrier injury model, Zhou et al. observed that HMGB1 disrupted endothelium integrity and increased endothelium permeability (184). Consistently, we observed that lactate promoted HMGB1 secretion *via* exosome release and induced endothelium barrier dysfunction (13). In addition, it has been stated that hyperglycemia is common in hospitalized COVID-19 patients and is strongly associated with worse outcomes (185–188). COVID-19 patients with early-onset hyperglycemia, defined as blood glucose > 180 mg/dl during the first 2 days after ICU admission, had higher levels of lactate than patients without hyperglycemia (186). In diabetes hyperglycemia promotes the release of HMGB1 and upregulates receptor for advanced glycation end products (RAGE) (189) (**Figure 4**). Notably, numerous studies show that HMGB1 facilitates thrombosis *via* promoting platelet activation and NET formation (190–192) (**Figure 4**). HMGB1 can also induce the expression and activation of tissue factor (TF), which is involved in inflammation-related thrombosis, in endothelial cells in a concentration dependent manner (193). Moreover, *in vitro* treatment of alveolar epithelial cells with exogenous HMGB1 increased the expression of SARS-CoV-2 entry receptor ACE2 (175) (**Figure 4**).

## Conclusions

SARS-CoV-2 infection causes metabolic reprogramming, such as increased glucose consumption and lactate production, which plays a role in the severity and mortality of COVID-19. Lactate is not only a valuable biomarker but also a critical signaling molecule in critical illness, including COVID-19. Thus, it is proposed that both reduced lactate production and inhibition of lactate-mediated signaling could improve COVID-19 (194). In this context, application of glycolysis inhibitors, such as 2-deoxy-D-glucose (2-DG), may have beneficial effects on COVID-19-infected patients. 2-DG is a glucose analogue which competitively inhibits the production of glucose-6-phosphate and consequently suppresses the glycolytic pathway (195). Our previous studies have demonstrated that at non-toxic dosages 2-DG markedly decreased lactate production and improved cardiac function in polymicrobial sepsis mice (196). Notably, the emergent use of 2-DG as an adjunct therapy in COVID-19 patients has been granted in India (197). In addition, animal studies show that lactate activates GPR81, a lactate specific receptor, to promote endothelial injury and immune cell dysfunction, which can be reversed by GPR81 inhibitors (12–14). Lactate can also be taken up by various cells through monocarboxylate transporter 1 (MCT1) (13, 198). We recently reported that blocking lactate influx by MCT1 inhibitor, as well as suppression of GPR81 signaling, decreased HMGB1 release from macrophages (13). Therefore, similar therapeutic strategies, either inhibition of lactate/GPR81 signaling or block lactate influx by MCT inhibitors, could also be used to abolish the detrimental effects of lactate in SARS-CoV-2 infection (194). On the other hand, priming the immune system with immunomodulatory components such as glucans may protect cardiovascular dysfunction in COVID-19. HMGB1 is a potential biomarker and may serve as a therapeutic target in severe COVID-19 (175). Our group previously reported that glucan phosphate improved cardiac function and suppressed HMGB1 translocation to the cytoplasm during sepsis (199, 200). This mode of action of glucan may counteract the effect of lactate in promoting HMGB1 release during SARS-CoV-2 infection (13). Importantly, a recent study has shown that glucans and mannans can be used as adjuvants to enhance the magnitude and durability of COVID-19 vaccines (201–203).

Since the outbreak of COVID-19, significant effort has been made to understand the pathogenesis of this new disease. With evidence collected from histological studies and biomedical tests, there has been increasing recognition that endothelial cell injury is one of the major contributors to the severity and mortality of COVID-19 infected patients. Recent evidence highlights the role of metabolism switching in the regulation of innate immune and inflammatory responses, which is observed in COVID-19 infected patients (204, 205). This review summarizes the potential role of aerobic glycolysis-derived lactate in the COVID-19 infection. In this mechanism of infection, lactate serves as an mediator that facilitates SARS-CoV-2 infection of endothelial cells, which leads to endothelial cell injury and multiple organ dysfunction. It is clear that growing evidence shows that lactate is involved in SARS-CoV-2-mediated endothelial cell death, vascular permeability, and coagulopathy. On the other hand, elevation of lactate levels, due to enhanced glycolysis, could also contribute to endothelial injury by altering immune cell function. Further basic science research is needed to validate whether targeting aerobic glycolytic metabolism could be beneficial for patients with COVID-19 infection.

## Author Contributions

KY, MH, MF, VL, YY were involved in the literature search, and drafting and preparation of the manuscript. KY, CL and XW were involved in the idea generation. KY, TH and DW were involved in checking the manuscript. All authors contributed to the article and approved the submitted version.

## Funding

This work was supported by National Institutes of Health grants HL071837 (CL), HL153270 (CL), GM083016 (CL, DW), GM119197 (DW), American Heart Association Postdoctoral Fellowship 916710 (MF), and C06RR0306551 (ETSU).

## Conflict of Interest

The authors declare that the research was conducted in the absence of any commercial or financial relationships that could be construed as a potential conflict of interest.

## Publisher’s Note

All claims expressed in this article are solely those of the authors and do not necessarily represent those of their affiliated organizations, or those of the publisher, the editors and the reviewers. Any product that may be evaluated in this article, or claim that may be made by its manufacturer, is not guaranteed or endorsed by the publisher.
